# Play behaviour, not tool using, relates to brain mass in a sample of birds

**DOI:** 10.1038/s41598-020-76572-7

**Published:** 2020-11-24

**Authors:** Gisela Kaplan

**Affiliations:** School of Science and Technology, University of New England, Armidale, NSW 2351 Australia

**Keywords:** Evolutionary developmental biology, Animal behaviour, Cognitive control, Sexual selection

## Abstract

Play behaviour and tool using in birds, two well-delineated and amply researched behaviours, have generally been associated with cognitive abilities. In this study, these behaviours were related to relative brain mass in a sample of Australian native birds. Despite suggestive research results so far between cognition and tool using, this study found no significant difference in relative brain mass or in lifespan between tool-using birds and non-tool users. By contrast, in play behaviour, subdivided into social players and non-social players, the results showed statistically very clear differences in relative brain mass between social, non-social and non-players. Social play was associated with both the largest brain mass to body mass ratios and with the longest lifespans. The results show that play behaviour is a crucial variable associated with brain enlargement, not tool using. Since many of the tool using species tested so far also play, this study suggests that false conclusions can be drawn about the connection between tool using and cognitive ability when the silent variable (play behaviour) is not taken into account.

## Introduction

This paper reports on an investigation of potential associations between relative brain mass and lifespan on one hand and two well-known and studied behaviours in birds, those of play behaviour and tool use, on the other. As its data base this paper deals exclusively with Australian native birds as a first overall examination of play behaviour of species native to this continent. In terms of the evolutionary base of modern songbirds and parrots this is of some importance since we know that modern songbirds and cockatoos and possibly other parrots have their origin in East Gondwana, now called Australia^1–3^. They either originated in the early Cretaceous period or first speciated within East Gondwana after the mass extinction event of 65 mya^4^. Play behaviour, particularly in many species of Australian parrots and cockatoos, is well-known, widespread and was possibly an established trait well before birds radiated to other continents about 30–20 million years ago, in the early Miocene^5^. With a few exceptions^6–9^, play behaviour in vertebrates has been far less well-studied in Australia than elsewhere.

Play behaviour in animals generally has nonetheless a long history in scientific research. Much excellent research has been undertaken in the manifestations of play as well as in the neural basis for play—the latter largely in mammals^10,11^. However, opinions are divided as to answering any of Tinbergen’s four questions about ultimate causes (adaptive value and evolution) as well as proximate causes (mechanism/causation and development) related to play behaviour. This is partly so because play behaviour is not ubiquitous and therefore, as a biological expression of a developing or adult organism, cannot be framed as a universal biological principle. It is possible, however, to reveal whether play behaviour is associated with other key biological and social criteria, some of which may belong to the neuro-cognitive domain^12^. The question emerging is in what way play behaviour may be of some benefit to an organism or enhance its overall fitness.

According to Burghardt’s definition of play behaviour^13^, animals from very different phyla, including even octopuses, turtles and cockroaches^14^, qualify for inclusion in play behaviour^15^. Evidence of play behaviour in birds is sprinkled unevenly across a number of avian orders but noticeably more concentrated in the order of Psittaciformes (parrots) and among Passeriformes (perching birds). The study here was undertaken to test play behaviour against relative brain mass and longevity in extant native birds whose ancestors never left the Australian continent.

As a comparison to play behaviour the category of tool using behaviour, a well circumscribed behaviour which at times has been considered a sign of complex cognition^16^, was also examined in relation to relative brain mass and to lifespan.

Play behaviour has been described and assessed in different ways and has led to many different hypotheses. Ironically, the clearest expression of its crucial importance in species that play has been found in cases in which opportunities for its expression are *absent*. Socially and play-deprived rats, for instance, have been shown to produce higher levels of stress hormones and to maintain these elevated levels for longer periods, exposing them to the detrimental effects of chronic stress, than do rats in socially and generally enriched environments^17,18^. Hence, it has always been tempting to hypothesise that animals that play are better prepared for adulthood. Play behaviour may have benefits for an organism^19^, be this in lowering stress^20^ or honing skills useful for adult life. Honing skills may be physical^21^, perceptual^22^ or social^23,24^. Alternatively, as has been argued, play behaviour may have little or nothing to do with future skills^25–28^. As Holekamp and Smale argued^29^, young animals should not be viewed as merely “imperfect adults with incomplete behavioural repertoires but rather as displaying behaviour that promotes their immediate competence and survival” and indeed, play behaviour is most often observed in young birds^30^. In other words, play behaviour has rather remained an uncomfortable category in which its potential functions have never been entirely satisfactorily explained.

Play behaviour is not universal. Not all mammals or birds show play behaviour. Worldwide examples of playing birds have been found in just 13 of the 40 orders and all published sources have been identified and documented in detail by Ortega and Bekoff^30^: there are published papers on species of the orders of Galliformes (grouse, quail), Anseriformes (ducks, geese etc.), Cuculiformes (cuckoos), Charadriiformes (puffins,shorebirds and relatives)^31^, Sphenisciformes (penguins), Pelecaniformes (ibis, herons, pelicans etc.), Strigiformes (owls), Bucerotiformes (hornbills, hoopoes etc.)^31^, Coraciiformes (kingfishers, bee-eaters etc.) and Falconiformes (falcons, caracaras). Among the most numerous and diverse number of families and species are the Psittaciformes (cockatoos, parrots, lorikeets)^33–36^ but outnumbering the parrots and all other orders by far are the Passeriformes (perching/songbirds)^9,34^, making up more than half of all extant birds. Diamond and Bond documented all known publications exclusively on social play, the most complex form of play and, worldwide, these have so far been found only within three orders (the third are the Bucerotiformes)^31^ an order not occurring in Australia. There is some overlap between Ortega & Bekoff^30^and Diamond & Bond^34^ references but both sources together provide a rather complete list of known play behaviour in birds and these references are therefore not reproduced here.

### Definition of what constitutes play behaviour and tool using

It is generally agreed that there are three distinct categories of play: single (or locomotory) play, object play and social play, and these categorisations have continued to be applied as meaningful categories in describing structure, and often context, of play behaviour^30^. Play fighting has quite often been treated and discussed separately, largely in small mammals^37,38^ and primates^20,39^. The descriptive play categories have been used for mammals and birds alike^40^.

In the first category, called solo, solitary or locomotory play, a very wide range of behaviours is included, such as running, skipping, jumping, ducking, rolling, hanging, swinging, dancing and even sliding and snow-romping as has been observed in common ravens^41^ and in keas, *Nestor notabilis*^42^, one of New Zealand’s parrots.

In the second category, object play, activities involve objects of any kind ranging from sticks, to stones and to small household objects^21,43–45^. Such object play in birds can lead to social play as a chasing game for the object that the first bird holds in its beak. That is, if such company of others is desired, the bird with the object might parade in front of others until they take the bait and join the game^46,47^. Even solo play can be contagious and get other birds to join in by performing the same activity (not necessarily interactively)—particularly noticeable in any form of sliding activities, be these performed by ducks using rapids as slides or the well-known snow-romping by keas and ravens^42,48^. Thus, both object and solitary play can turn into communal or social play^34,50^.

The third category is social play and involves the playing together of any two or more individuals. Such interactions have been documented in far fewer species than solo or object play. Published cases so far tend to be largely limited to psittacines^8,35,49^ and corvida, such as magpies, ravens^7,9,41,50,51^, and very occasional even in raptors, such as Montagu’s harrier^52^. Social play may include aggressive and play-fighting on the ground^37,38^ and in the air^9^. In muroid rodents^53^, targeting for a fight might have grown out of amicable behaviour (e.g., social investigation, greeting, allo-grooming) rather than, as may be the case in other taxa, from agonistic behaviour and thus may have multiple precursors^53^.

Tool using is known to be present and has been studied in a wider range of species than play behaviour^54^. Some raptors have become well-known for their tool or proto-tool use, as it has in Lammergeiers, feeding on bone-marrow and taking large bones into the sky to drop them on rocky outcrops and thus splintering the bones to a size on which the birds can feed^55^, or in many sea birds and crows dropping shelled invertebrates such as clams and shelled molluscs^56–58^. True tool use is seen in the black-breasted buzzard, releasing rocks from their beaks to crack emu eggs^59^ or the black kite picking up burning ambers and twigs and transporting them to dry grass areas, which promotes the burning of substrate and allows the bird to feast on fleeing or scolded insects and vertebrates^60^. Among the most spectacular and well-documented cases of tool use in psittacines and corvids are the New Caledonian crow with its varied tool use and tool manufacture behaviour^61^ and the very unusual behaviour of drumming with a stick in palm cockatoos^62,63^.

### Rationale for this study

Graham and Burghardt (2010) reviewed the field of play behaviour^64^ and suggested to biologists to be bold and to take seriously the challenges that play behaviour provides in terms of evolution and general applicability for survival. One can equally argue that it is premature to even consider play behaviour in an overall cognitive framework and speak globally about the phenomenon of play behaviour in birds given that only a miniscule fraction of species of the oriental, neotropical and Australo-Asian and oceanic bird regions of the world has been included in any assessment of play behaviour so far, with some very notable exceptions^42,61,62,65,66^. In fact, in those aforementioned regions, very few avian species have been studied in any detail in their natural environment for their behavioural repertoires, let alone for play behaviour. Yet these barely discussed regions, are actually the bird-rich regions of the world and would likely produce a yet almost untapped wealth of new material. Even anecdotal but published evidence of play behaviour of wild birds in the Southern hemisphere is still relatively rare and, with the exception of the parrots of New Zealand^35,42^ and some birds in Australia^9,66^ and the very occasional nod in the direction of neotropic, tropical or oriental psittacines, often on problem-solving tasks rather than play behaviour^67–70^, what we know of the very playful behaviour of many psittacine species is often the result of observations of captive birds. Studies of captive birds may provide a glimpse into the potential richness of the field of play behaviour but captivity creates its own rules for survival and some observed behaviours may be artefacts of captivity.

However, as we finally have details of brain weights of Australian psittacines and perching birds^71,72^ there is now an opportunity to bring play behaviour and tool using together with brain mass and investigate whether there are trends emerging that may covary or even be causally related. It is of particular interest from an evolutionary point of view. To reiterate: East Gondwana (now Australia) is the cradle of modern songbirds^1,2^ and some identified extant psittacine species have an evolutionary history going back to the Cretaceous on this continent^3^.

This study here tests the hypothesis that large-brained birds play more or, vice versa, those that play more have larger brains, a question that has been asked for some time^73,74^. This hypothesis has some support in the study of various mammals showing that brain sizes and life outcomes vary according to individual and social conditions^75–77^. It has been shown that play can have a substantial impact on brain size and learning^10^ and that domestication, in fact, can modulate rat behaviour of different strains in various ways, such as in play-fighting^78^.

Iwaniuk et al.^73^ who linked play behaviour and brain size used three orders—rodents, marsupials and primates. They found conflicting evidence for the hypothesis that large-brained species could reliably predict play prevalence and complexity. However, their research also showed that orders with, on average, larger brains were more likely to contain species that are more playful^73^.

Another theme to which the Discussion below will refer is the link between length of maturation and the incidents of play behaviour and brain size^74^, discoveries and suggestions that are now a good two decades old but have so far rarely been followed up in species of the continent (Gondwana/Australia) in which modern passerines and some psittacines are known to have first evolved.

The hypothesis formulated here has built on existing data concerning brain mass, lifespan and play behaviour on one hand^15,40^ and brain mass and tool use on the other^16,54^. Stated summarily, it is hypothesised that play behaviour is associated with large brain mass relative to body mass and also with longevity. Since tool using is also thought to be associated with large brain size, as shown in well-documented studies of large-brained individual species^79^ and both, play behaviour and tool using have been linked with heightened cognition generally^80^, in this study, tool using has been included as an additional parameter.

## Results

### Play behaviour

#### Play behaviour and lifespan

**Figure 1:**
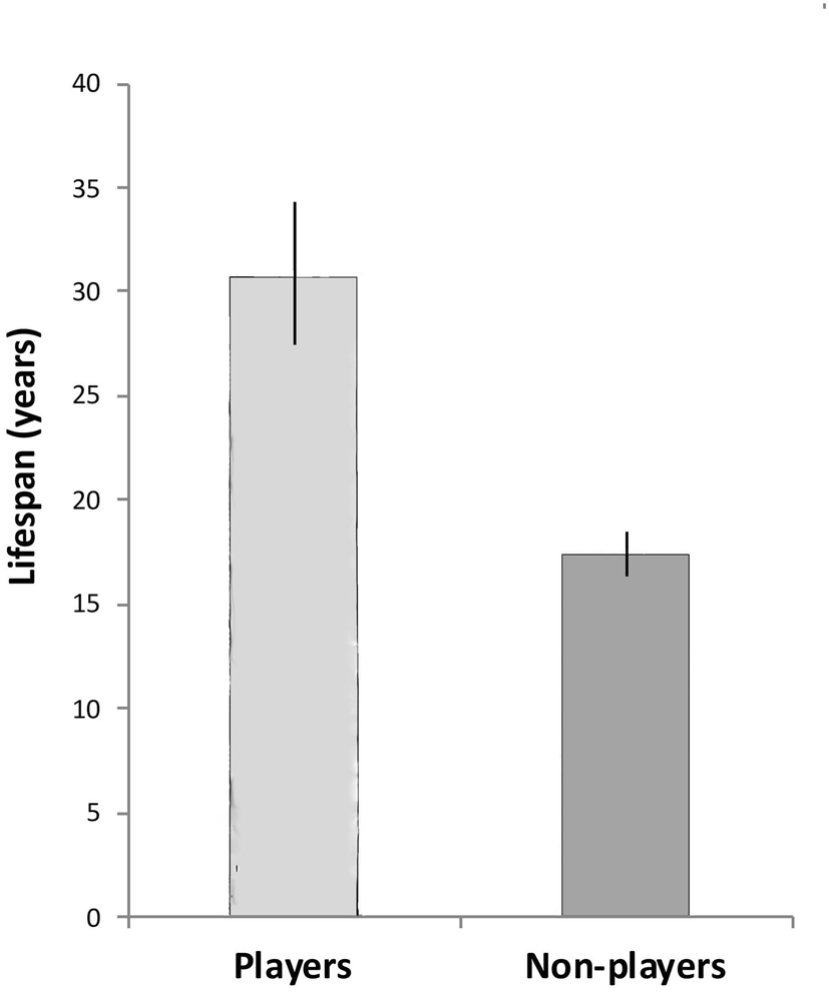
Lifespan (in years) of players (N = 35) compared to non-players (N = 42). Means and standard errors are presented. Note that species that play live twice as long as those that do not play (*p* = 0.0007, see text for details).

First, a comparison was made between the lifespans of species known to perform play of any kind (N = 35) and lifespans of those species that do not play (N = 42). Results revealed that players have significantly longer lifespans than non-players (mean ± sem for players = 35.21 ± 3.68 years, and for non-players 17.24 ± 1.15 years; 2-tailed *T* test: *t* = 5.0138, df = 75, *p* = 0.0001; see Fig. 1).

**Figure 2:**
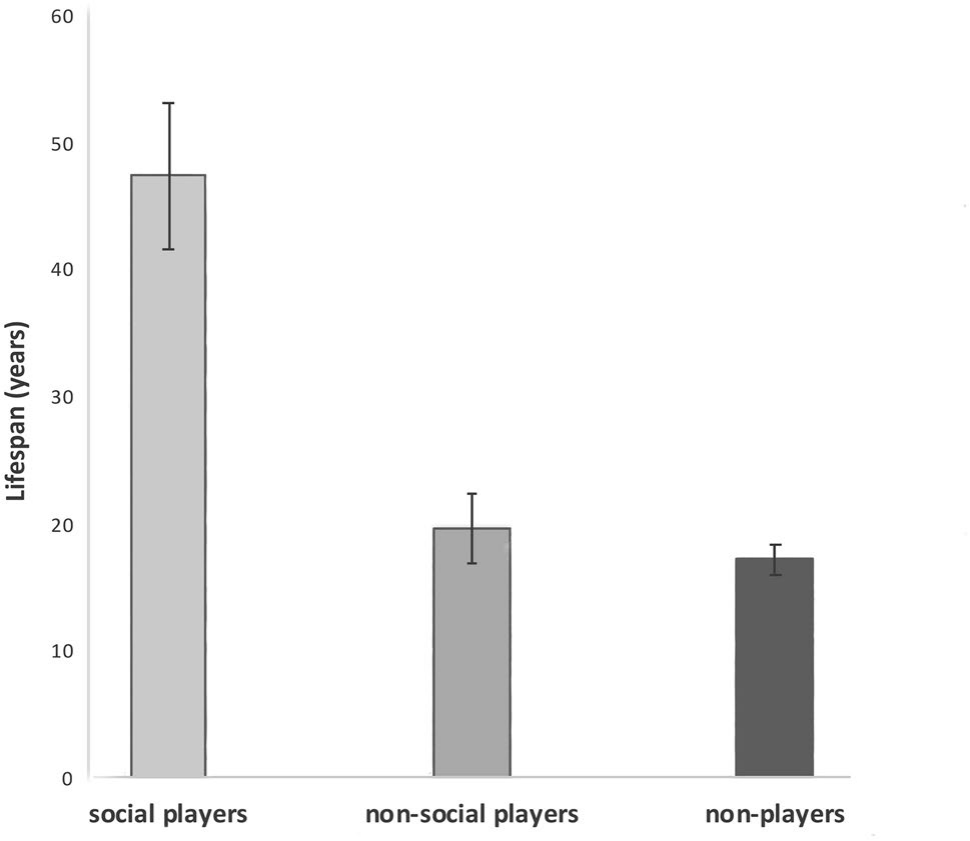
Lifespan associated with type of play. Means and standard errors are presented. Lifespan is substantially and significantly longer only in those species that perform social play. The lifespans of species that perform non-social play do not differ from those of the species that do not play.

Next, species that play were separated into those known to perform social play (N = 19) versus those that perform only other, non-social play (N = 16), see Fig. 2. For reasons of sample size, solo and object play were put together as one category of ‘non-social’ play. A one-way ANOVA of these groups (social players, non-social players, non-players) was significant (F _(2,75)_ = 30.474_,_ *p* > 0.00001). Subsequent T-tests showed that the association between lifespan and play applies only to social playing (mean ± sem of lifespan of social players is 47.47 ± 5.74 years, and of non-social players it is 19.69 ± 2.71 years: 2-tailed *T* test, *t* = 4.1201, df = 33, *p* = 0.0002; Fig. 2). There is no significant difference in lifespan between non-social players and the group that performs no play of any kind, non-players (2-tailed *T* test: *t* = 0.334, df = 56, *p* = 0.3304).

#### Play behaviour and relative brain mass

**Figure 3:**
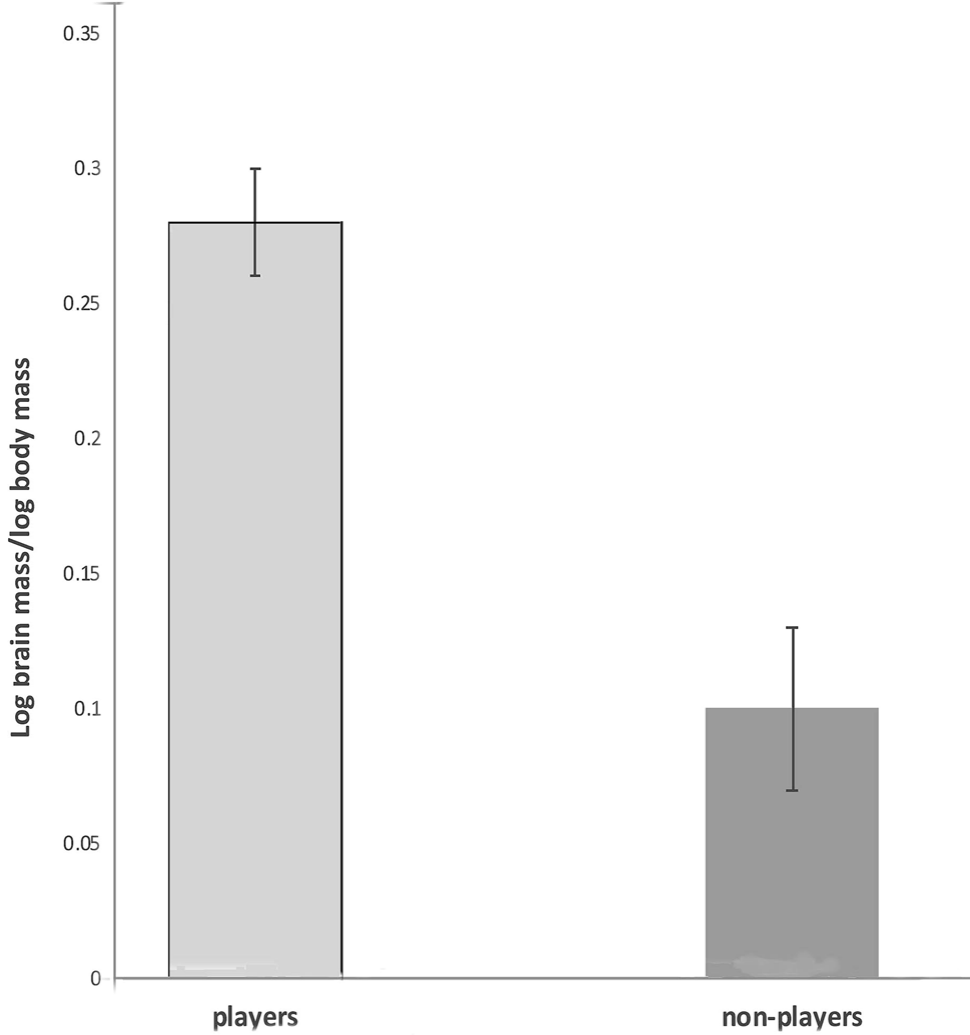
Log of brain mass relative to log of body mass in species that perform play versus those that do not play. The difference is significant (*p* = 0.0001; see text for details).

Brain mass relative to body weight (log_10_ brain mass/log_10_ body mass) was compared between the species that performed any type of play (players: N = 35) and the species that do not play (N = 42). For players the mean size of this ratio ± sem was 0.28 ± 0.02 and for non-players it was 0.10 ± 0.03. The difference is significant (2-tailed *T* test, *t* = 4.7854, df = 75,*t* = , *p* < 0.0001): players had significantly larger relative brain mass compared to non-players (Fig. 3).

This effect of play on relative brain mass was examined further by analysing the data for parrots separately from that of other species. For parrots alone, the ratio of the log brain mass to log body mass was significantly greater in players (mean 0.328 ± 0.020) than in non-players (0.223 ± 0.043) (T = 2.2502, df = 25, 2-tailed *p* = 0.0335). Similarly, for the other species (i.e., not parrots) the ratio was higher for players (1.219 ± 0.034) than for non-players (1.085 ± 0.035) (T = 2.1368, df = 49, 2-tailed *p* = 0.0376).

The issue of relative brain size and absolute brain size was raised by Matějů et al.^81^, who found little support for a link between sociality and relative brain size in an order of mammals but showed that sociality increased with absolute brain size and also with body mass. Based on their result, absolute brain mass and body mass in players versus non-players in the avian sample were also compared. Although there was no difference between these two groups in body mass (means and sems, players 380.9 ± 38.6 g, and non-players 403.2 ± 128.6 g; 2-tailed *t* test, df = 75, *t* = 0.1534, *p* = 0.87), absolute brain mass did differ significantly (players 7.26 ± 0.82 g, non-players 3.58 ± 0.58 g; 2-tailed *t* test, df = 75, *t* = 3.4831, *p* = 0.0008). Hence, independent of body mass, brain mass is larger in those avian species that play compared to those that do not play and this applies both to parrots (mean brain mass with sem of players is 9.00 ± 1.24 g and for non-players it is 3.72 ± 1.09 g; T = 1.963, df = 25, 2-tailed *p* = 0.006) and to species other than parrots (2.47 ± 0.11 g for players compared to 1.98 + 0.12 g for non-players; T = 2.327, df = 49, 2-tailed *p* = 0.024).

**Figure 4:**
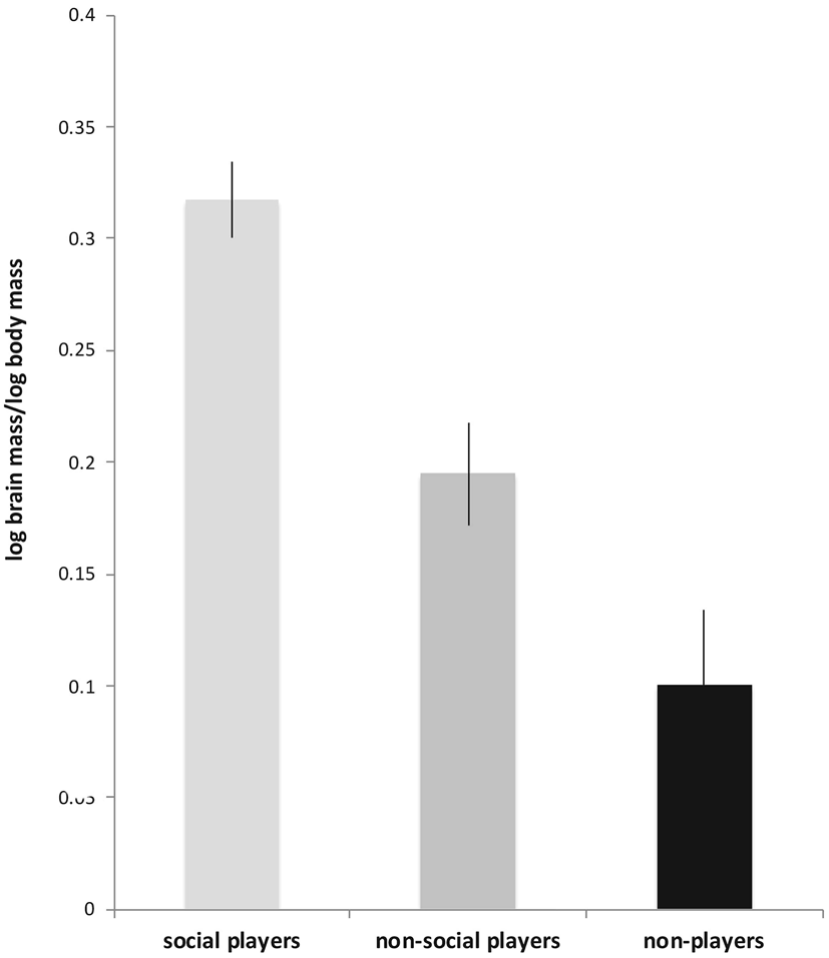
Type of play is associated with relative brain mass. Relative brain size is highest for social players, but even non-social players (solo or object play) have significantly larger brains than non-players.

After separating the players into social players and non-social players, the full data set (i.e. including non-players) was analysed by one-way ANOVA and found to be significant (F_(2,76)_ = 13.101, *p* = 0.000013). Comparison of the ratio of the log brain mass to to log body mass ratio of those species that are known to play socially (N = 19) versus those known to play but do so only non-socially (N = 16) revealed that this ratio was significantly larger for social players than for non-social players (2-tailed *T* test: *t* = 3.4912, df = 33, *p* = 0.0014; Fig. 4). Social play goes with having a larger brain to body weight ratio.

Furthermore, the brain to body weight ratio of non-social players compared to non-players was also significant (2-tailed *T* test: *t* = 2.2335, df = 56, *p* = 0.0295), indicating that non-social playing also increases the mass of the brain relative to body mass but to a lesser degree than does social play.

## Tool using and life span

The data set was divided into tool users (N = 26) versus non-tool users (N = 48) in order to examine a possible association between tool using, longevity and relative brain size.

**Figure 5:**
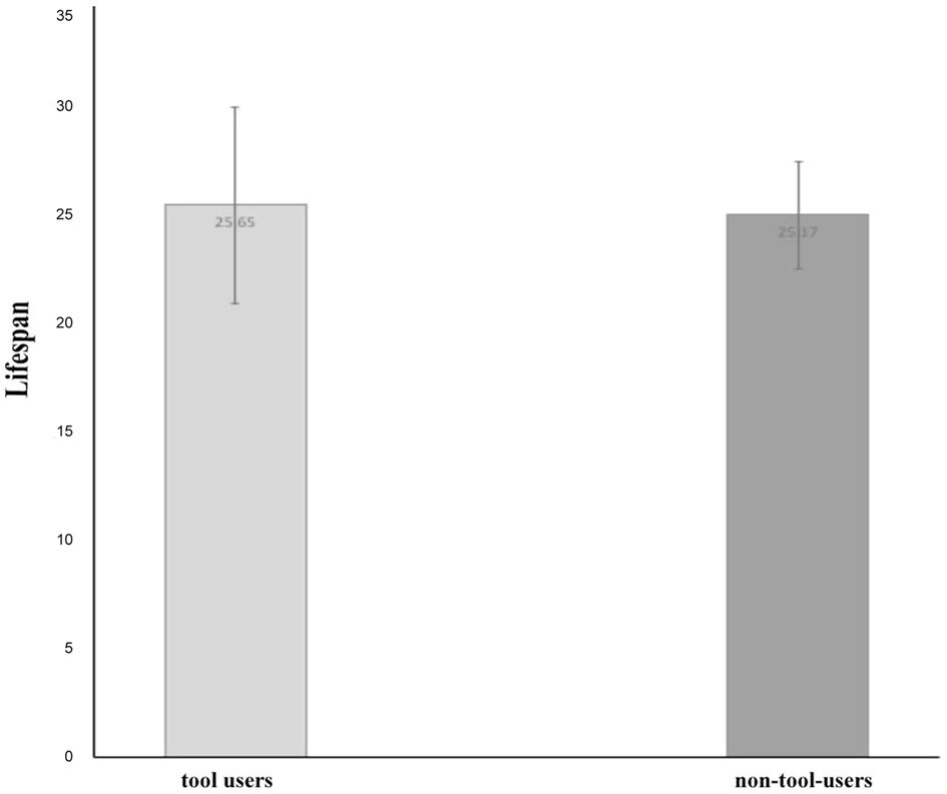
Mean life span in years with standard errors is presented for tool users (N = 26) and non-tool users (N = 48). There is no significant difference between these two groups.

Comparison of the lifespan of tool users versus non-tool users revealed no significant difference in lifespan. The tool users live a mean ± sem of 25.65 ± 4.57 years compared to 25.17 ± 2.47 years for the non-tool users (2-tailed *T* test: *t* = 0.1002, df-74, *p* = 0.9205; Fig. 5).

### Tool using and relative brain size

**Figure 6:**
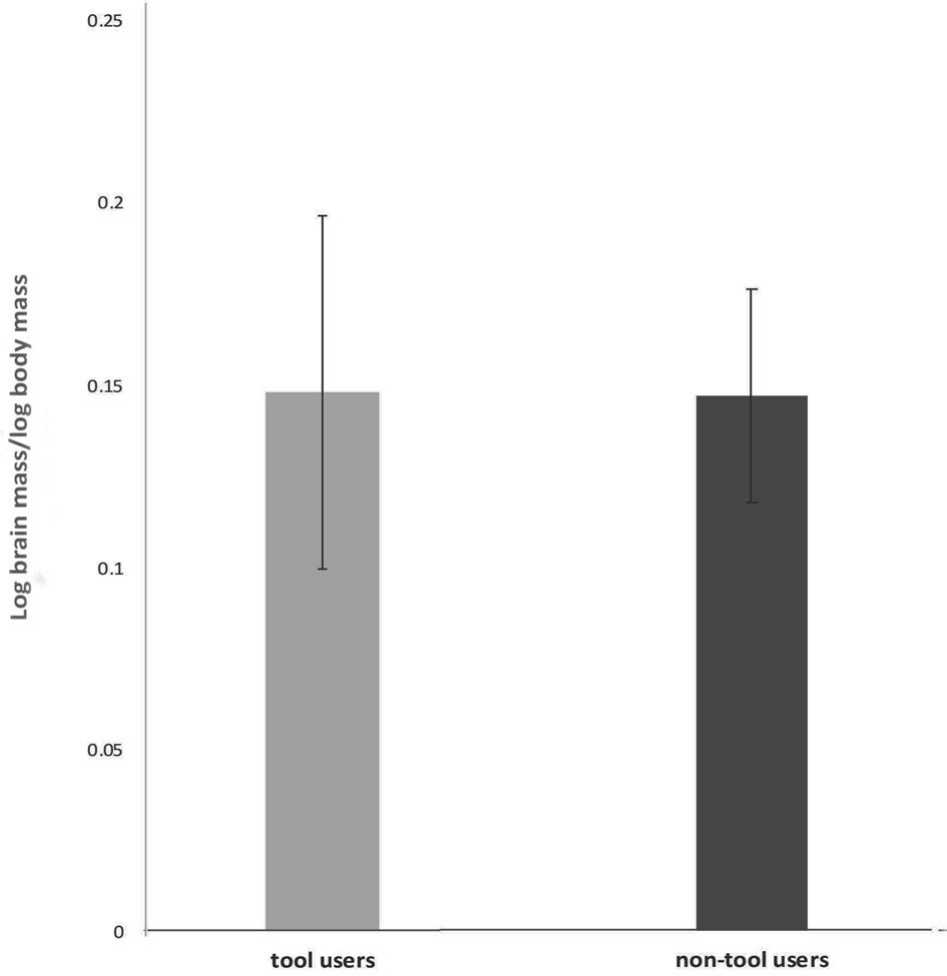
Tool using compared to brain size. Tool use has no bearing on relative brain mass. Means with standard errors are plotted.

In this sample there was no significant difference between tool users and non-tool users in relative brain size. The log of brain mass/log of body mass is 0.198 ± 0.046 (mean ± sem) for tool users and 0.174 ± 0.027 for non-tool users (2-tailed *T* test: *t* = 0.4712, df = 74, *p* = 0.6389; Fig. 6).

## Discussion

This study analysed data from 5 to 7 orders of birds indigenous to Australia for play behaviour, as against brain mass, lifespan and tool using. A significant association between lifespan and play behaviour (Fig. 1) was found but only for social players and not for non-social players (Fig. 2). It also showed that players had larger brains than non-players (Fig. 3). Surprisingly, even non-social players (solo or object play) were shown to have significantly larger brains than non-players, but not as large as social players. The difference was also significant (Fig. 4).

Since the strongest evidence of the association of play with large brains was found in species that play socially, it required further testing. Social play, as said before, has been identified in Australian species in just two orders, albeit the most numerous, the Psittaciformes and the Passeriformes. To avoid a potential conclusion that this significant difference in brain mass between players and non-players in all categories could be biased in favour of parrots and cockatoos, known for their extensive play behaviour, the parrots were separated from non-parrots and both groups (Psittaciformes and Passeriformes) were analysed separately from the same data set. The results showed that the differences in ratio of the log brain mass to log body mass remained significant for either group, stronger in parrots than in songbirds, but nevertheless significant in both orders. In other words, forms of play were associated with a larger brain. The comparisons of lifespan and tool use, however, yielded no significant results at all. Lifespan was comparable whether a species used tools or not (Fig. 5). Even more surprising was the finding that tool using and non-tool using birds showed no differences in relative brain mass (Fig. 6).

Play behaviour may thus be a significant factor in juvenile development for large-brained birds while it raises questions about the link between tool using and brain mass. The results here are exciting in that both types of engagement in play, be it social or non-social (solo or object) play, were found to have a direct association with relative brain mass. Even non-social players showed greater brain mass than non-players. Most importantly, relative brain mass was much larger in social players compared to brain mass in species that do not engage in play behaviour.

Most play behaviour occurs during adolescence and, in Australian species, is typically part of a long-drawn out maturational process. Indeed, this immature stage post-fledging generally takes up a fifth of the entire lifespan of large-brained birds and, as in other large-brained vertebrates, provides an extended period for brain development. Importantly, periods to sexual maturity or readiness for establishing a pair relationship and breeding can be delayed by 4–5 years^82,83^ as in white-winged choughs^84^, in Australian magpies^9^ and even in bowerbirds in which males have to wait for 7 years before being competitive and some cockatoo species take up to 7 years to reach sexual maturity^85,86^.

The protracted development also means that parents have to take an active role of supervision and protection. Indeed, more than 60 percent of Australian birds receive a minimum of 50 days of close parental supervision and care post-fledging and often much longer, as compared to 18 percent of avian species in high latitudes^82,83^. This prolonged protection creates the conditions safe enough for self-involved play of youngsters as well as for brain development.

The literature is clear about the benefits of play behaviour in terms of stress reduction as measured by levels of the stress hormone, be this in mammals (cortisol) or in birds (corticosterone)^87^. This is one benefit that is instantaneous and as Pellis and Pellis had argued, is ‘playing for the present’^75^. High levels of corticosterone impede growth, also of the brain, cause cell and plumage damage and ultimately also shorten the lifespan^90,91^. Conversely, lifespan extensions and larger brain mass have been linked to the absence or reduction of stress. In birds, Lendvai et al*.*^92^ found that those species with larger brains relative to their body size, at any stage in their life history, had a lower baseline and lower peak levels of the stress hormone corticosterone than species with smaller brains^92^. Hence, reducing or eliminating stress in a juvenile bird by playing could thus have a direct effect on life expectancy, success in breeding and a number of other life-history events.

Oddly, social play with its thrills and mock risks (as in hide-and-seek, chasing and play-fighting games) clearly also creates its own stress. A stimulus that is detected as a physical challenge or is perceived as threatening becomes a stressor when the hypothalamic–pituitary–adrenal (HPA) axis is activated^93^. In early welfare studies in chickens, it was found that fear could be modulated and reduced by providing environmental enrichment^94^. Playing, especially play fighting, activates the same neuro-chemical pathways^12,95,96^ but then somehow intercepts these pathways. In other words, the individual brain learns to interpret the stressor as harmless and can bring stress levels down very swiftly. Learning to control stress levels and reducing levels of corticosterone quickly thus becomes a valuable adaptation when facing real risks and dangers. Playing leads to faster reaction time and overall stress reduction *at the time* of playing for which there is ample evidence in rodents^95^.

Social play has so far been found in just three orders of birds worldwide (Psittaciformes, Passeriformes and Bucerotiformes), as mentioned before, and in just two orders in Australia. Indeed, social players make a much smaller group than that found if all solo and object playing species worldwide are considered. However, this still represents taxonomically up to nine separate families in the Australian sample. One notices that species so far shown to engage in social play behaviour with relative brain masses well above the mean of Australian land birds, either belong to psittacines or to a specific and relatively large cluster of birds that can be described as the Corvoidea within the order of Passeriformes, a superfamily to which drongos, fantails, mud-nesters, birds of paradise, monarchs, flycatchers , shrikes, Australian magpies and butcherbirds, and all the many species of crows and ravens belong^40^. The species within this super family are thus of special interest in a wider and specifically in an evolutionary sense.

This study reported here suggests that play behaviour is not at all marginal or a fringe benefit but part of an evolutionary development in a specific cluster of birds. As the results have shown, social play is clearly identifiable as being associated with overall brain mass and thus it seems to confirm the social brain hypothesis^97^. Whether social play is a consequence of the social brain^98,99^ or whether social play behaviour is causally related to growing a larger brain cannot be decided on the basis of these results and will require much further research over a wider group of species observed and studied in the natural environment.

**Figure 7:**
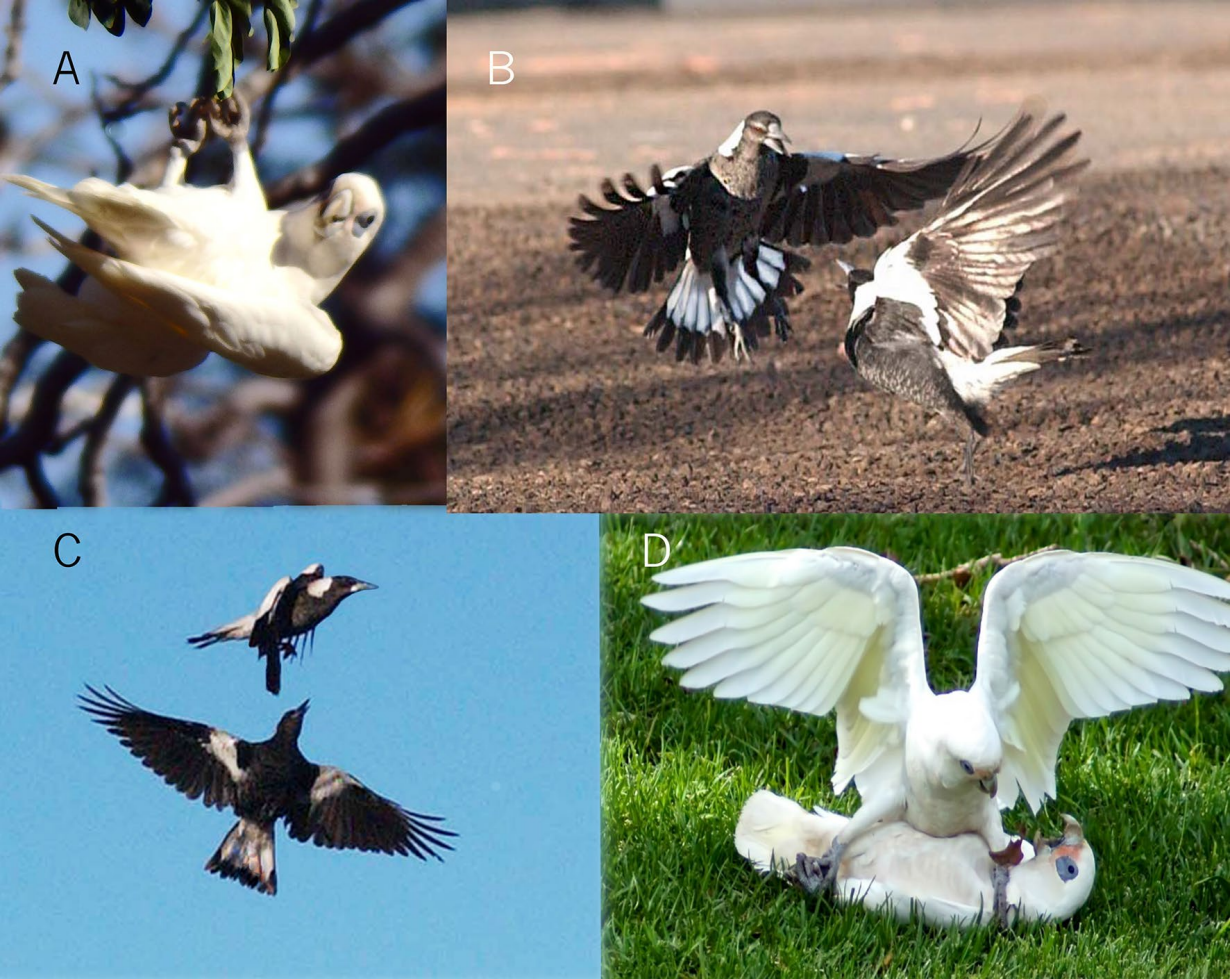
Four different play contexts. (**A)** Solo play by little corella (*Cacatua sanguinea*)-holding on to small hanging branch with both feet, the bird swings from side to side as fast and as far as it can, as if on a swing. (**B)** Social play-wild Australian magpies *Gymnorhina tibicen*; juveniles at play (identifiable as juveniles by scalloped grey belly feathers) in mutual intimation and ramming attempts conducted close to the ground-can turn into vigorous play-fighting. (**C)** Aerial play fighting/chasing by juvenile magpies: the aim of the pursuer is to get hold of a tail feather and destabilise the bird in front. (**D)** Object play of one little corella turning into play-fighting: Note the small dark spot on the left leg of the dominant bird (a desiccated leaf). The other bird had ‘owned’ that leaf but the dominant one tried to snatch it and, in that attempt, subdued the original holder of the leaf. Note that the bird lying on its back has clasped the right foot of the bird above, giving it a hold on the dominant bird. They can wrestle by locking on to the opponent’s foot and then roll around for a wrestling match. Often, the original prize is entirely forgotten and a few seconds or minutes later they feed peacefully next to each other. (Credits: A, B, and C by author; D: Bobbi Marchini).

The question keeps being asked what constitutes social complexity and social intelligence in birds^100^. It seems that play behaviour offers very good and discrete sets of interactions to test specific cognitive abilities and undertake neuroethological studies to test the relationship of brain activity and brain growth, memory and other factors against play activities. Such studies are still rather sparse even in human children and adolescents. A rare study by Peskin and Ardino^101^ found that playing hide-and-seek is conceptually quite a difficult task: few 3-year-old , not even all 4-year old children are able to play this game successfully, yet juvenile Australian magpies play this game extensively. They also play-fight and play fight in flight^9^ (Fig. 7).

An interesting paper by Smaldino et al.^102^ examines how complexity in play behaviour (i.e. in social play) interacts with behavioural variation and socio-cognitive demands. In their mathematical modelling the authors arrived at the conclusion that the evolution of complex play is favoured when the maximum benefit of complex play is high and when the additional investment needed to manifest that benefit is low. There is a problem, however: additional investment appears to be particularly high in cockatoos and some passerines in the sense that prolonged/delayed maturation, an environment in which play behaviour can thrive and reach substantial sophistication, requires enormous additional investment by the parent birds. Perhaps it can be argued that high survival rates of offspring and longevity are substantial benefits that still outweigh such investments, a point that González‐Lagos et al. conceded a decade ago^103^. They found that, at least in mammals, there is support for the hypothesis that the costs of affording large brains have been counterbalanced with a longer reproductive life^103^.

Finally, the comparison group of tool-using birds showed perhaps the greatest surprise. It seems that within the available sample of native birds no extra brain capacity (or, at least, brain mass) is needed at all in order to use tools! Intuitively, this runs counter to expectations. Evidence of tool using behaviour or proto-tool use, at least in cockatoos and parrots, in the natural environment is usually restricted to observations of twig manipulations and has not been well described other than anecdotally. Palm cockatoo tool use in the wild, as an exception, has been well-researched. Palm cockatoos may break twigs to the right length to furnish the bottom of the nest hole as an inserted platform (to avoid water logging of the nest) and the male uses sticks to drum in territorial and courtship displays and may even carry a favourite stick to other roosting sites^63^.

Tool use is typically tested in the laboratory, as it has in the Goffin’s cockatoo, also called Tanimbar corellas (*Cacatua goffiana*) at home on the island of Tanimbar north–north west of Australia and part of the Australian plate. In captivity, they have been shown to be able to deal with tools and they are very adept at opening complex locks on par with the performance of chimpanzees^104^ and New Caledonian crows as well some cockatoos even manufacture them^61,63,105^. In the captive context, Goffin’s cockatoos have indeed performed exceptionally well^105^. There is a problem however. A recent study of the behaviour of wild Goffin’s cockatoos, possibly the first comprehensive study of its kind in this species and undertaken by the same laboratory^106^, found no evidence of tool use whatsoever in the wild. The birds obviously have little need to engage in this behaviour in the wild -they do not use tools in the wild.

The authors suggest that the tool-related capacities of Goffin’s cockatoos may “stem from domain general processing, which, in turn, could represent an adaptation to opportunistic extractive foraging”^106^. It has long been recognised that play behaviour may lead to tool use^107^ and that the origins of play behaviour (and indeed of cognitive complexity) can be associated with relative complexity of their social groups and/or environment^108,109^. In other words, tool use is a potential ability that may be an adaptation to a complex or, as the case may be, very restrictive feeding niche while play behaviour might have an important function in development.

Tool using has been linked with complex cognition or a link has been implied in many studies, from mammals to birds. The problem lies partly in the modular research approach to behaviour that one distinct type of behaviour is singled out and then tested against relative brain mass. Tool-using has tended to be investigated on its own and conclusions have been drawn from displayed scale and complexity of that one specific set of behaviour at the exclusion of other possible contributing cognitive candidates that could explain the link to brain size.

Here it has been shown that, at least in the sample of Australian birds, there is no association between tool-using and relative brain mass. The point here is to propose that those documented outstanding performances in tool use and even tool use manipulation and technical know-how in some avian or mammalian species might reveal that the species tested with the greatest abilities in tool use may also be shown to have well-established patterns of play behaviour. One key methodological problem may lie not in the nature of comparative studies but in the exclusion of ‘silent’ variables such as play behaviour that may substantially distort results obtained for tool-using behaviour.

To test this assumption, one would need to compare more non-playing/tool-using birds with non-playing/non-tool using birds. I am aware of only one study in which a non-playing tool-using species, the Galápagos woodpecker finch, *Camarhynchus pallidus*, was compared in its performance with a non-tool-using (nonplaying) relative, the small tree finch, *Camarhynchus parvulus*^110^, and this has shown that one species did not outperform the other in cognitive tasks.

The results here do not underplay the importance and, at times, sophistication of tool using in birds but it is a matter of whether these are adaptations or predispositions and what dynamic interaction may be cause or consequence or covariation. It has been argued that different cognitive abilities may belong to ‘different cognitive building blocks’^106^. Likewise, specific abilities can be co-opted to serve new purposes^111^. Showing no relationship between tool use and relative brain mass as has been the case in this study, however, might well require some further reflection. Innovative behaviour, problem solving and tool using may be indicative of exploratory behaviour indeed and some of it may become very sophisticated^112^ but this is likely not to be the lynch-pin.

Play behaviour, especially social play, ticks all the boxes as an evolutionary driver for brain size increase, bringing with it physiological, hormonal and metabolic changes as well as the ability for close social attention to others, be these companions, coalitions or opponents in play fights. As Pellis and Pellis argued so eloquently, complex play including play fighting, involves subtle signals between play partners that are tactical and thus suggest a reliance on cognitive processes “involving attention, cue assessment and perhaps some planning”^88,89^. The counter-argument has been at times that developing a large brain (presumably for increased cognitive functionality) is biologically very costly but has been counterbalanced by lengthening lifespans and reproduction. It is increasingly recognised that the same principle may apply to some avian species.

Relative brain mass and play behaviour have been shown here to have a consistent and close association, both in the wild and in captivity, since different types of play, be this non-social play (solo, object) or social play, are reflected in relative brain size, and for social play, even in absolute brain size^81^.

Relative brain size as a measure of cognitive abilities is a hotly debated topic about mosaic (in parts) or concerted (whole) evolution of the brain^112,113^. We now have evidence that the number of neurons in the avian forebrain (pallium) correlates with measurements of cognition^114^ and that the brains in large-brained birds so far examined, such as parrots and songbirds, contain on average twice as many neurons as primate brains of the same mass. Moreover, so Olkowicz et al. discovered, corvids and parrots have much higher proportions of brain neurons located in the pallial telencephalon compared with primates or other mammals and birds^115^.

The emphasis in this paper, though, has been to examine the link between a number of behavioural variables and the dynamics of these variables that might contribute to brain mass and its likely presence of cognitive abilities, already in evidence for a number of large-brained birds such as cockatoos and parrots^116^, keas^117^ and corvids^118^. All of the species tested in great detail for their cognitive abilities, one might add, are either extant birds of the southern hemisphere with Gondwanan origins or, as in corvids, have distant Gondwanan heritage.

Ancient Gondwanan lineages of extant modern songbirds show a high level of cooperative breeding^119^ and of female song^120^, two attributes that have almost been lost once species had radiated to other continents. By contrast, play behaviour seems more than merely a superficial social behaviour but a biological adaptation found amongst a wide variety of mammals including humans, with similar outcomes as described here for birds. Another similar evolutionary trait that now seems confirmed is a causal link between large brain size and lifespan^121,122^, usually also meaning a slowing down of reproduction and a maturational delay for each generation. These findings place the results on play behaviour here into a consistent and important biological pattern rather than among incidental and superficial behavioural traits. If this result is sustainable not just at the place of origin of modern birds, i.e. East Gondwana, but elsewhere in the world, it is very possible that in the birds examined here, play behaviour is both a mechanism and an outcome a) of their foraging niche, b) of the way they are raised and c) an evolutionary progression that has seen those species adapt to coping with vastly changing climate and feeding conditions over the millions of years on the Australian continent.

## Methods

Information gathering: A data set was assembled that included relative brain sizes (brain mass), lifespan, and confirmation or not of any play behaviour and tool use. A full list of all Australian land-birds with these and other measures and life history details can be found in the Appendix of *Bird Minds*^40^ which also includes data of relative brain size of Australian land birds, i.e. of some 500 species, based on Franklin et al.^72^, used as a basis for the final sample size in this study. Franklin et al. data include native, non-vagrant species of the Australian mainland and its continental islands and their set comprises data on the brain mass and body mass of 504 species representing all Australian avian orders (encompassing 74.6% of the Australian avifauna.) and they sampled 96.2% of Psittaciformes and 71.1% (224 species) of Australian passerine species. Their brain volume measurements were converted into mass and for the relevant species examined in this paper they had measured brain volume/mass for more than one specimen, arriving at mean brain volumes/mass which are the measures used in this paper.

The actual orders, families and species used and referred to in this study are listed below and for each behaviour separately. Likewise, tool using behaviour and data for life spans were derived from published accounts (see under source material below).

### Sample size

The sample size was originally 145 but almost half had to be discarded because either lifespan or type of play data were not complete or unavailable. Only those species for which lifespan data could be obtained were included in the samples that were analysed. This sample included 77 species. The full sample was then subdivided into players (N = 35) versus non-players (N = 42). Of the species that play, it was possible to make a subdivision between those that play socially (N = 19) and those that play but not socially (N = 16). A second subdivision was made on the basis of reliable evidence of tool using (N = 26) versus non-tool using species (N = 48).

In summary, species were included of which lifespan data with relevant information exhibiting play and/or tool using behaviour and completely logged brain versus body mass data were available. Players included in the analysis belonged to 5 orders and 9 families (see below). The non-players belong to 9 orders and 22 families. Tool-users included in the analysis belonged to 5 orders and 9 families (see below). The non-tool users belong to 9 orders and 22 families. Amongst the players, 34.5% are also tool users and amongst non-players 24% are also tool users. Details of species included in the analysis for both categories of play and tool use are listed below. Notably, there would have been plenty of choice to use a wide variety of non-players and non-tool users among the entire range of Australian songbirds. The only constraint has been as to whether lifespan data were available. Species in which lifespan data were patchy or non-existent were excluded.

The data were collated and data points assessed (i.e. relative brain mass versus lifespan, play behaviour or tool use). Tool using was used as a comparison a) because of its concrete delineations of physical interaction with a tool and b) because tool using has been associated with innovative behaviour, problem-solving and generally with more complex cognitive abilities (16).

The data for three treatment groups (non-players, social players and non-social players) were analysed first by using one-way ANOVAs and then by using 2-tailed *T* test comparisons with Bonferroni correction for multiple comparisons (α = 0.025 for lifespan and log_10_ brain mass to log_10_ body mass comparisons).

Full bibliographical details of publications on Australian native avian players and tool users are listed below, called ‘Source material’.

#### Brain data and compilation of behavioural data

The main source for brain and body weight data is Franklin et al. (Ref.^72^ below) and the Appendix in Kaplan (Ref.^40^ below) which includes Franklin et al. and, in addition, offers life history details such as lifespan, play, tool using, innovative behaviour, parenting models) for all Australian land birds.

### Species used for analysis of play behaviour and tool use (Australian species only)

Orders are underlined, followed by common and Latin species names.

#### Species Used Showing Play behaviour (in any category), by order and family

Accipitriformes. Accipitridae: Black kite, *Milvus migrans*. Columbiformes: Columbidae (pigeons and doves): topknot pigeon, *Lopjolaimus antarcticus*. Psittaciformes: Cacatuidae (cockatoos): Palm Cockatoo, *Probosciger aterrimus;* Red-tailed Black-Cockatoo, Calyptorhynchus banksia; Yellow-tailed Black-Cockatoo, Calyptorhynchus funereus; Sulphur-crested Cockatoo, *Cacatua galerita*; Pink Cockatoo, *Cacatua leadbeateri*; Galah, *Cacatua roseicapillus*; Long-billed Corella, *Cacatua tenuirostris*; Western Corella, *Cacatua pastinator*; Little Corella, *Cacatua sanguinea,* Cockatiel, *Nymphicus hollandicus*. Psittacidae (lorikeets and parrots): Rainbow Lorikeet, *Trichoglossus haematodus*; Princess parrot, *Polytelis alexandrae*; Crimson Rosella, *Platycercus elegans*; Eastern Rosella, *Platycercus eximius*; Pale-headed Rosella, *Platycercus adscitus*; Northern Rosella, *Platycercus venustus*; Budgerigar, *Melopsittacus undulates*; Bourke's Parrot, *Neopsephotus bourkii*. Coraciiformes: Dacelonidae (kookaburras and allies): Laughing Kookaburra, *Dacelo novaeguineae.* Passeriformes: Ptilonorhynchidae (bowerbirds), Satin bowerbird, *Ptilonorhynchus violaceus*. Corcoracidae (mud nesters): White-winged chough, *Corcorax melanorhamphos*; Apostlebird, *Struthidea cinerea*; Passeriformes: Artamidae (magpies, butcherbirds, currawongs and allies): Australian magpie, *Gymnorhina tibicen.* Passeriformes: Corvidae (ravens and crows): Australian raven, *Corvus coronoides*; Torresian Crow, *Corvus orru.*

#### Species used showing no play behaviour

Galliformes: Megapodiidae (megapodes): Australian Brush-turkey, Alectura latham; 2-Accipitriformes: Accipitridae: Grey Goshawk, Wedge-tailed Eagle. 3-Falconiformes: Falconidae: Brown Falcon; Australian Hobby. Columbiformes: Columbidae (pigeons and doves): Bar-shouldered dove, *Geopelia humeralis.* Psittaciformes, Cacatuidae (cockatoos): Carnaby's black-cockatoo; Baudin's Black-Cockatoo, Gang-gang Cockatoo. Psittaciformes: Psittacidae (lorikeets and parrots). Musk Lorikeet, *Glossopsitta concinna;* Purple-crowned Lorikeet, Double-eyed Fig-Parrot, Eclectus Parrot, Regent Parrot, Western rosella, Red-capped Parrot, Turquoise parrot, *Neophema pulchella.* Caprimulgiformes: Podargidae (frogmouths): Tawny frogmouths, Podargus strigoides. Coraciiformes: Dacelonidae (kookaburras and allies): Rainbow bee-eater, Dollarbird. Passeriformes: Menuridae: superb lyrebird. Passeriformes: Motacillidae (wagtails and pipits): Grey wagtail, *Motacilla cinereal;* Passeriformes: Petroicidae (Australasian robins): Red-capped robin, *Petroica goodenovii*; Easter yellow robin, *Eopsaltria australis.* Passeriformes: Pachycephalidae (whistlers, shrike-thrushes and allies): golden whistler, *Pachycephala pectoralis*; grey shrike-thrush, *Colluricincla harmonica.* Passeriformes: Monarchidae (monarchs, flycatchers and magpie larks): restless flycatcher, *Myiagra inquieta*; willi wagtail, *Rhipidura leucophrys*, Magpie lark, *Grallina cyanoleuca*; Passeriformes: Pomatostomidae (babblers): Hall’s babbler, *Pomatostomus halli*. Passeriformes: Maluridae (wrens): Superb fairy-wren, *Malurus cyanneus.* Passeriformes: Acanthizinae (scrubwrens, thornbills and gerygones): White-browed scrubwren, striated thornbill, brown thornbill. Passeriformes: Climacteridae (treecreepers): white-throated treecreeper, brown treecreeper. Passeriformes: Meliphagidae (honeyeaters and Australian chats): Little Wattlebird, *Anthochaera lunulate*; Lewin’s honeyeater, *Meliphaga lewinii;* yellow-faced honeyeater, *Lichenostomus chrysops*; yellow-tufted honeyeater*, Lichenostomus melanops*; Passeriformes: Oriolidae (orioles and figbirds): Figbird, *Sphecotheres vieilloti*; regent bowerbird, *Sericulus chrysocephalus*. Passeriformes: Artamidae (magpies, butcherbirds, currawongs and allies): grey butcherbird, *Cracticus torquatus*; Passeriformes: Zosteropidae (white-eyes): Silvereye. Passeriformes: Ploceidae/Estrildidae (finches): Zebra Finch, *Taeniopygia guttata,* red-browed finch, *Neochmia temporalis.*

#### Species used displaying tool using behaviour

Galliformes: Megapodiidae (megapodes): Australian Brush-turkey, Alectura latham. Accipitriformes: Accipitridae: Black kite, *Milvus migrans.* Wedge-tailed eagle*, Aquila audax.*, Eastern osprey, *Pandion cristatus*. Psittasiformes*.*Cacatuidae (cockatoos): Palm Cockatoo, *Probosciger aterrimus*;Long-billed corella*, Cacatua tenuirostris;* Sulphur-crested Cockatoo, *Cacatua galerita;* Psittacidae (lorikeets and parrots): Rainbow Lorikeet, *Trichoglossus haematodus;* Musk Lorikeet, *Glossopsitta concinna;* Budgerigar, *Melopsittacus undulates;* Turquoise parrot, *Neophema pulchella.* Caprimulgiformes: Podargidae (frogmouths): Tawny frogmouths, *Podargus strigoides*. Coraciiformes: Dacelonidae (kookaburras and allies): Laughing Kookaburra, *Dacelo novaeguineae.* Passeriformes; Monarchidae (monarchs, flycatchers and magpie larks): Magpie lark, *Grallina cyanoleuca.* Passeriformes: Maluridae (wrens), superb fairy-wren, *Malurus cyanneus.* Passeriformes: Meliphagidae (honeyeaters and Australian chats): Lewin’s honeyeater, *Meliphaga lewinii*. Passeriformes: Corcoracidae (mud nesters): White-winged chough*, Corcorax melanorhamphos*. Passeriformes: Artamidae (magpies, butcherbirds, currawongs and allies): Black-faced Woodswallow, *Artamus cinereus*. Passeriformes: Oriolidae (orioles and figbirds): Satin bowerbird, *Ptilonorhynchus violaceus*; Spotted Bowerbird, *Ptilonorhynchus maculatus*; Green Catbird , *Ailuroedus crassirostris*. Passeriformes: Corvidae (ravens and crows): Torresian Crow, *Corvus orru.* Passeriformes: Ploceidae/ Estrildidae (finches): Red-browed Finch, *Neochmia temporalis.* Passeriformes: Acanthizinae (scrubwrens, thornbills and gerygones): Yellow-rumped Thornbill, *Acanthiza chrysorrhoa .*

#### Species used that are non- tool users

Accipitriformes: Accipitridae Grey Goshawk (*Accipiter novaehollandiae*), Falconiformes: Falconidae: Brown Falcon (*Falco berigora*), Australian Hobby (*Falco longipennis*), Columbiformes: Columbidae (pigeons and doves): bar-shouldered dove (*Geopella humeralis*). Psittaciformes: Cacatuidae (cockatoos): red-tailed black-cockatoo (*Calyptorhynchus banksii* ), yellow-tailed black-cockatoo (*Calyptorhynchus funereus* ), Carnaby's black-cockatoo (*Calyptorhynchus latirostris*), Baudin's Black-Cockatoo (*Calyptorhynchus baudinii* ), gang-gang cockatoo (*Callocephalon fimbriatum*), pink cockatoo (*Cacatua leadbeateri*), galah (*Cacatuas roseicapillus*), western corella (*Cacatua pastinator*), little corella (*Cacatua sanguinea*), cockatiel (*Nymphicus hollandicus*). Psittacidae (lorikeets and parrots): Purple-crowned lorikeet (*Glossopsitta porphyrocephala*), Eclectus parrot (*Eclectus roratus*), regent parrot (*Polytelis anthopeplus*), princess parrot (*Polytelis alexandrae*), crimson rosella (*Platycercus elegans*), Eastern rosella (*Platycercus eximius*), pale-headed rosella (*Platycercus adscitus*). Coraciiformes: Dacelonidae (kookaburras and allies): Rainbow Bee-eater (*Merops ornatus*), Dollarbird (*Eurystomus orientalis*), Passeriformes: Menuridae: Superb Lyrebird (Menura novaehollandiae). Climacteridae (treecreepers): white-throated treecreeper (*Cormobates leucophaeus*), brown treecreeper (*Climacteris picumnus* ). Passeriformes: Meliphagidae (honeyeaters and Australian chats): Little wattlebird (*Anthochaera lunulata*, yellow-faced honeyeater (*Lichenostomus chrysops* , yellow-tufted honeyeater (*Lichenostomus melanops*), fuscous honeyeater (*Lichenostomus fuscus*) , New Holland honeyeater (*Phylidonyris novaehollandiae*), figbird (*Sphecotheres vieilloti* ), Passeriformes: Ptilonorhynchidae (bowerbirds): regent bowerbird (*Sericulus chrysocephalus*), Passeriformes: Pachycephalidae (whistlers, shrike-thrushes and allies): Golden Whistler (*Pachycephala pectoralis*), Grey Shrike-thrush (*Colluricincla harmonica*) . Passeriformes: Corcoracidae (mud nesters): Apostlebird (*Struthidea cinerea* ). Passeriformes: Monarchidae (monarchs, flycatchers and magpie larks): restless flycatcher (*Myiagra inquieta*), willi wagtail (*Rhipidura leucophrys*), Passeriformes: Pomatostomidae (babblers): Hall's babbler (*Pomatostomus halli*), Passeriformes: Acanthizinae (scrubwrens, thornbills and gerygones): white-browed scrubwren (*Sericornis frontalis*), striated thornbill (*Acanthiza lineata*), brown thornbill (*Acanthiza pusilla*). Passeriformes: Zosteropidae: Silvereye (*Zosterops lateralis*).

### Source materials

#### Lifespan data

##### Internet sources

AnAge: The animal ageing and longevity database: https://genomics.senescence.info/species/.

Bioacoustics: https://bioacoustics.cse.unsw.edu.au/archives/html/birding-aus/2002-04/msg00467.html.

Australian Bird Study Association (ABSA). Bird in the Hand.(2nd edition). https://absa.asn.au.

Geering, D. Longevity of Aust birds. Attachment to ‘birding-aus@vicnet.net.au 24 April https://bioacoustics.cse.unsw.edu.au/archives/html/birding-aus/2002-04/msg00467.html (2002).

Longevity Records: https://www.demogr.mpg.de/longevityrecords/0303.htm; www.demogr.mpg.de.

##### Publications

Brouwer, K., Jones, M.L., King, C.E. & Schifter, H. Longevity records of Psittaciformes in captivity. *Intern. Zoo Yearbk* **37** (1), 299–316 (2000).

#### Taxonomy

Gill F, D Donsker & P Rasmussen (eds). IOC World Bird List (v10.2), 2020. doi : 10.14344/IOC.ML.10.2.

The subdivision of Australian songbirds into corvida (and the superfamily of corvoidea) and passerida is based on Sibley, C.G. & Ahlquist, J.E. Phylogeny and Classification of Birds: a Study in Molecular Evolution. (Yale University Press 1990)-for more detailed information see Kaplan (Ref.^40^).

### Access to published sources for observed avian play behaviour and tool using in Australian species not cited in references

Chisholm, A. H. *Bird Wonders of Australia.* Sydney. (Angus and Robertson, 1948).

Chisholm, A. H. The use by birds of "tools" or "instruments". *The Ibis* **96**, 380–383. (1954).

HANZAB: Handbook of Australian, New Zealand & Antarctic Birds.

-Vol. 4. *Parrots to Dollarbird.* Ed. PJ Higgins. Melbourne (OUP, 1999).
-Vol. 5. *Tyrant-flycatchers to Chats*. Eds. PJ Higgins PJ and WK Steele. (OUP, 2001).
-Vol. 6. *Pardalotes to Shrike-thrushes*. Eds. PJ Higgins and JM Peter. (OUP 2002).
-Vol. 7. Part A. *Boatbill to Larks*. Eds. PJ Higgins and SJ Cowling. (OUP 2006a).
-Vol 7. Part B. *Dunnock to Starlings*. Eds PJ Higgins, JM Peter and SJ Cowling. (OUP 2006b).
